# Characteristics and Predictors of Death among Hospitalized HIV-Infected Patients in a Low HIV Prevalence Country: Bangladesh

**DOI:** 10.1371/journal.pone.0113095

**Published:** 2014-12-08

**Authors:** Lubaba Shahrin, Daniel T. Leung, Nashaba Matin, Mohammed Moshtaq Pervez, Tasnim Azim, Pradip Kumar Bardhan, James D. Heffelfinger, Mohammod Jobayer Chisti

**Affiliations:** 1 Dhaka hospital, International Centre for Diarrheal Disease Research, Bangladesh (icddr,b), Dhaka, Bangladesh; 2 Centre for Nutrition and Food Security (CNFS), International Centre for Diarrheal Disease Research, Bangladesh (icddr,b), Dhaka, Bangladesh; 3 Centre for HIV/AIDS (CHIV), International Centre for Diarrheal Disease Research, Bangladesh (icddr,b), Dhaka, Bangladesh; 4 Centre for Vaccine Sciences (CVS), International Centre for Diarrheal Disease Research, Bangladesh (icddr,b), Dhaka, Bangladesh; 5 Royal London Hospital, Barts Health NHS Trust, London, United Kingdom; 6 Global Disease Detection Branch, Division of Global Health Promotion, Center for Global Health, Centers for Disease Control and Prevention (CDC), Atlanta, Georgia, United States of America; FIOCRUZ, Brazil

## Abstract

**Background:**

Predictors of death in hospitalized HIV-infected patients have not been previously reported in Bangladesh.

**Objective:**

The primary aim of this study was to determine predictors of death among hospitalized HIV-infected patients at a large urban hospital in Bangladesh.

**Methods:**

A study was conducted in the HIV in-patient unit (Jagori Ward) of icddr,b's Dhaka Hospital. Characteristics of patients who died during hospitalization were compared to those of patients discharged from the ward. Bivariate analysis was performed to determine associations between potential risk factors and death. Multivariable logistic regression was used to identify factors independently associated with death.

**Results:**

Of 293 patients admitted to the Jagori Ward, 57 died during hospitalization. Most hospitalized patients (67%) were male and the median age was 35 (interquartile range: 2–65) years. Overall, 153 (52%) patients were diagnosed with HIV within 6 months of hospitalization. The most common presumptive opportunistic infections (OIs) identified were tuberculosis (32%), oesophageal candidiasis (9%), *Pneumocystis jirovecii* pneumonia (PJP) (8%), and histoplasmosis (7%). On multivariable analysis, independent predictors of mortality were CD4 count ≤200 cells/mm^3^ (adjusted odds ratio [aOR]: 16.6, 95% confidence interval [CI]: 3.7–74.4), PJP (aOR: 18.5, 95% CI: 4.68–73.3), oesophageal candidiasis (aOR: 27.5, 95% CI: 5.5–136.9), malignancy (aOR:15.2, 95% CI: 2.3–99.4), and bacteriuria (aOR:7.9, 95% CI: 1.2–50.5). Being on antiretroviral therapy prior to hospitalization (aOR: 0.2, 95% CI: 0.06–0.5) was associated with decreased mortality.

**Conclusion:**

This study showed that most patients who died during hospitalization on the Jagori Ward had HIV-related illnesses which could have been averted with earlier diagnosis of HIV and proper management of OIs. It is prudent to develop a national HIV screening programme to facilitate early identification of HIV.

## Background

As of 2012, an estimated 35.3 million people were living with human immunodeficiency virus (HIV) globally and among them 2.3 million were newly infected [1]. In the Asia Pacific region, the estimated number of new HIV infections was 350,000 (220,000–550,000) and the number of AIDS-related deaths across the region was 270,000 (190,000–360,000) [2]. By the end of 2013, the Ministry of Health and Family Welfare (MOHFW) of Bangladesh had documented 3,241 persons with confirmed HIV, including 1,299 persons who developed AIDS and 472 persons who had died [3]. With an estimated HIV prevalence of <0.1% in the general population [3], Bangladesh has been considered a low HIV prevalence country [4] since the first case was detected in 1989 [5]. Despite these low numbers, Bangladesh is one of the nine countries in the world in which the estimated HIV incidence increased by >25% between 2001 and 2011 [6].

Diagnosis of HIV in Bangladesh is often delayed for a variety of reasons, including lack of awareness of HIV infection due to its low prevalence, the limited number of HIV testing and counseling facilities, which is often a critical entry point for engagement into care and prevention, and the unwillingness of many people to be tested [7], [8]. As a result, HIV infected persons are diagnosed in late stages of the disease and most hospitalized patients with HIV have a CD4 count of <200 cells/mm^3^ [9]. The Dhaka Hospital of the International Centre for Diarrhoeal Disease Research, Bangladesh (icddr,b) opened the Jagori Ward, an inpatient unit for the management and treatment of people living with HIV (PLHIV), in May 2008. The experience from the Jagori Ward's first 21 months (from May 2008 to February 2010) has been previously reported; we found that 38% of those who died had active tuberculosis (TB) and that the majority of patients admitted to the ward had a CD4 cell count <50 cells/mm^3^ [9]. However, we were unable to determine independent risk factors associated with death due to low sample size and the limited number of variables collected. Little information is available regarding the clinical presentation of AIDS in Bangladesh and there is no literature on predictors of mortality among HIV-infected persons hospitalized in Bangladesh. The primary objective of this study was to determine independent predictors of death during hospitalization. A secondary objective was to estimate the proportion of deaths among those hospitalized on the Jagori Ward. Such data could provide critical information about severity of HIV in PLHIV and thus lead to improvement of the clinical management of PLHIV in this low-prevalence country.

## Materials and Methods

### Study design

This was a retrospective observational study conducted from February 2009 –December 2012. Data were collected after extraction of electronic medical records of patients hospitalized on the Jagori ward. Patient's information was anonymized and de-identified prior to analysis. This study (protocol number: PR-12078) was approved by icddr,b's Research Review and Ethical Review Committees. It also received approval from the Centers for Disease Control and Prevention, USA, as not involving human subject research.

### Study setting

Dhaka Hospital's Jagori Ward has provided in-patient services for HIV-infected patients and served as a major referral center for PLHIV since May 2008. Currently, it admits over 100 PLHIV annually. In February 2009, Dhaka Hospital implemented an electronic medical-record system whereby patients are provided a unique patient identification number on each admission. All personal and clinical data collected are recorded in this access-protected medical record system. The majority of patients admitted to the Jagori Ward are referred by peer-led non-governmental HIV care and support groups (hereafter referred to as nongovernmental organizations or NGOs), and the most of the remaining patients were referred from government and private hospitals. If not already engaged with an NGO, patients are introduced to NGOs prior to discharge and the NGOs are then responsible for providing antiretroviral (ARV) medication and primary care to the patients. Following medical record extraction of variables described below, all identifiable patient's information was deleted from the research database.

### Study population

All confirmed HIV-infected patients who were admitted to the Jagori Ward during the study period were enrolled in this study. Data were collected only for first admissions during the study period and included data obtained until discharge or death during that admission, though information about deaths during subsequent hospitalizations in the course of the study period were collected. Unique study identification numbers were generated to distinguish re-admission and follow-up visits. For this analysis, we only used data collected from patients that were entered into the electronic medical record system.

### Study definitions

During admission, a trained counselor obtained social histories from each patient and entered these into their medical records. The self-reported route of HIV transmission, self-reported high-risk sexual behaviors (including having multiple sex partners, sex with commercial sex workers, or male-male sex), receipt of blood and blood products, injection drug use, and for children, having a parent with HIV infection, were recorded. Information on migrant work history was obtained. Migrant work history was defined as history of work outside of Bangladesh by patients or their spouses or by parents of pediatric patients. By the term “HIV-related conditions” we understood characteristics consistent with World Health Organization (WHO) stage IV AIDS defining illnesses/infection [10]. Clinical staging of HIV and management of opportunistic infections (OIs) were performed according to WHO guidelines [10], [11].

Diagnostic criteria for presumptive OIs were as follows: TB – clinical features suggestive of TB with characteristic radiological features and/or smear-positive for acid-fast bacilli; *Pneumocystis jirovecii* pneumonia (PJP) – suggestive clinical features including hypoxemia (SaO_2_ <90%) [12], CD4 cell count <200 cells/mm^3^, bilateral, diffuse interstitial infiltrates on chest radiograph, and/or high lactate dehydrogenase enzyme (LDH) in the absence of TB or fungal infection [10]; histoplasmosis – characteristic umbilicated skin eruptions with prolonged fever and/or features of bone marrow suppression with CD4 cell count <100/mm^3^ and high LDH and/or detection of fungal elements consistent with *Histoplasma capsulatum* by histology from skin, lymph nodes, or bone marrow [10]; oesophageal candidiasis – creamy white, plaque-like lesions extending beyond the oropharynx with odynophagia, anorexia, or burning sensation in the throat with CD4 cell count <200/mm^3^ [11]; cytomegalovirus infection (retinitis, colitis, oesophagitis) – GI symptoms (bloody diarrhea, abdominal pain, colitis) or visual impairment and characteristic fundoscopy features and CD4 cell count <50/mm^3^ [10]; cerebral toxoplasmosis – features of encephalitis with focal neurological signs and multiple lesions in neuroimaging of brain and/or positive anti-toxoplasma immunoglobulin G (IgG) and CD4 cell count <100/µl [11]; cryptococcal meningitis – features of meningitis with CD4 cell count <100/µl and cerebrospinal fluid findings suggestive of meningitis with suggestive neuroimaging [10], [11].

### Data management and analysis

The following variables were extracted from the medical records of Jagori Ward patients: age, gender, exposure history, duration of HIV diagnosis, presenting symptoms, physical examination findings, WHO staging at discharge/death, AIDS-defining illnesses, nadir and most recent CD4 cell counts, taking ARV medications at the time of admission, and taking co-trimoxazole at the time of admission. For deceased patients, duration of hospitalization was recorded and causes of death were extracted from death certificates and for the discharged patients duration of hospitalization and diagnosis on discharge were extracted from the discharge certificate of the same medical records. All data were entered into a database (SPSS version 17.0; Chicago, IL, USA). Differences in proportions were compared by the Chi-square test or Fisher-Exact test as appropriate. Differences in means were compared by Student's t test (for normally distributed data) or Mann-Whitney test (for data that were not normally distributed). P-values less than 0.05 were considered statistically significant. Strength of association was determined by calculating adjusted odds ratios (aOR) and their 95% confidence intervals (CIs).

In identifying independent factors associated with deaths in patients, variables were initially analyzed using a bivariate model, then independent predictors were identified using a logistic regression model using a backward, stepwise approach beginning with inclusion of all variables significantly associated with death on bivariate analysis (p-value ≤0.05) and including only those variables with p-values ≤0.05 in the final model.

## Results

**Figure 1:**
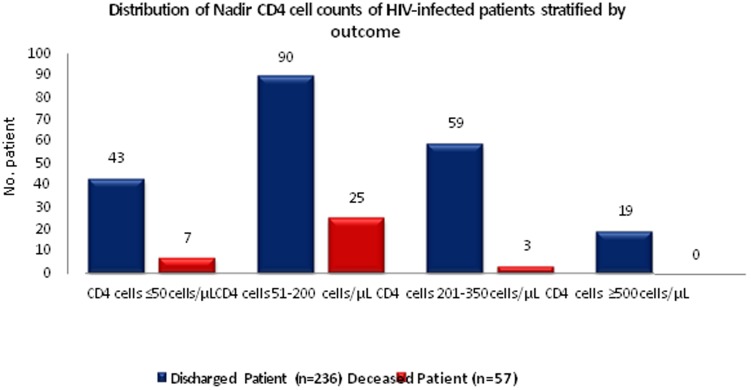
Distribution of Nadir CD4 cell counts of HIV-infected patients stratified by outcome.

We reviewed 651 hospital admission records of the Jagori Ward by HIV-infected individuals during February 2009 to December 2012; this included admissions of 293 unique and 358 repeat admissions by these individuals. Of the 293 unique patients admitted to the Jagori Ward, there were 57 (19%) deaths during the first visit. Sixty-seven percent of the hospitalized patients were male and the median age was 35 (range: 2–65) years. The median duration of hospitalization was 6 (interquartile range: 1–87) days. Seventy-seven percent of patients had a history of migrant work. A total of 24 (8%) of patients reported a history of injection drug use. Comparison of baseline characteristics and laboratory findings for deceased and discharged patients are shown in Table 1. The nadir CD4 cell counts and WHO stages of deceased and discharged patients are shown in Fig. 1.

**Table 1:** Baseline characteristics of the HIV-infected patients stratified by outcome.

Characteristic		Deceased (n = 57)	Discharged (n = 236)	OR (95% CI)	P-value
**Gender**					
	Male (%)	40 (70)	155 (66)	1.2 (0.6–2.4)	0.62
	Female (%)	17 (30)	81 (34)		
**Age in years, median (range)**		36 (9–60)	35 (2–65)	----	0.19
**Exposure history**					
	High-risk sexual exposure	47 (83)	193 (82)	1.0 (0.4–2.4)	0.89
	Injection drug user	7 (12)	17 (7)	0.5 (0.2–1.57)	0.21
	Mother-to child transmission	1 (2)	21 (9)	5.4 (0.7–111.5)	0.06
	Blood product	1 (2)	4 (2)	0.9 (0.1–23.1)	0.97
	Unknown	0	2(1)	undefined	
**History of migrant work**					
	Migrant worker	33 (58)	120 (51)	0.75 (0.4–1.4)	0.33
	Spouse of migrant worker	7 (12)	46 (20)	1.73 (0.7–4.5)	0.2
	Child of migrant worker	1 (2)	16 (7)	4.07 (0.5–84.1)	0.14
	None (no relation with migrant work)	16 (28)	54 (23)	0.76 (0.4–1.5)	0.4
**New HIV diagnosis** (<6 months prior to admission)		38(67)	115(49)	**2.1(1.1–4.0)**	**0.02**
**WHO clinical staging on admission**					
	Stage I	0	52 (22)	undefined	0.001
	Stage II	0	79 (34)	undefined)	0.001
	Stage III	9 (16)	79 (34)	2.7 (1.2–6.2)	0.001
	Stage IV	48 (84)	26 (11)	0.02 (0.01–0.06)	<0.001
**Nadir CD4 cell count (≤200/mm ^3^)**		54 (95)	130 (55)	**14.5 (4.4–60.6)**	**<0.001**
**Current ART use**		23 (40)	145 (67)	**0.3 (0.2–0.6)**	**<0.001**
**Most recent CD4 cell count/mm^3^ (median, IQR)**		47 (3–236)	161 (5–1549)	----	<0.001
**Haemoglobin (gm/dl) (median, IQR)**		7.3 (2.8–13.8)	10.30 (2.7–15.90)	-----	<0.001
**Total white cell count(1000/mm^3^) (median, IQR)**		6.5 (0.50–42.11)	6.0 (0.88–37.84)	-----	<0.54

Figures represent n (%), unless specified. OR: odds ratio. CI: confidence interval. IQR: interquartile range.

Diagnoses stratified by outcome are shown in Table 2. Among the 93 patients with presumptive TB diagnoses, 48 (53%) had pulmonary TB, 5 (6%) had TB meningitis and the rest had various other forms of extrapulmonary TB. A total of 11 patients were diagnosed with malignancies: four had non-Hodgkin's lymphoma, two had central nervous system tumours, and one each had Kaposi's sarcoma, mediastinal sarcoma, cervical cancer, carcinoma of the tongue and adenocarcinoma of the colon. The mean nadir CD4 cell count was lower for patients who died (69±60 cells/mm^3^) than for those who were discharged (223±216 cells/mm^3^). *Cytomegalovirus* infection, cerebral *Toxoplasmosis, Cryptococcal* meningitis and *Herpes* viral infection were not associated with death on bivariate analysis.

**Table 2:** Diagnoses of HIV-infected patients stratified by outcome.

Clinical condition	Deceased	Discharged	OR (95% CI)	P-value
	(n = 57)	(n = 236)		
**Presumptive tuberculosis**	26 (46)	67 (28)	**2.1 (1.1–3.9)**	**0.02**
**Presumptive ** ***Pneumocystis jiroveci*** ** pneumonia**	15 (26)	8 (3)	**10.2 (3.8–28.2)**	**<0.001**
**Presumptive histoplasmosis**	12 (21)	9 (4)	**6.7 (2.5–18.6)**	**<0.001**
**Presumptive oesophasial candidiasis**	15 (26)	12 (5)	**6.7 (2.7–16.5)**	**<0.001**
**Malignancy** *	5 (9)	6 (3)	3.7 (0.9–14.3)	0.04
***Presumptive Cytomegalovirus*** ** infection**	4 (7)	11 (5)	1.5 (0.4–5.5)	0.5
**Presumptive Cerebral ** ***Toxoplasmosis***	2 (4)	1 (1)	8.6 (0.5–242.6)	0.09
**Presumptive ** ***Cryptococcal*** ** meningitis**	1 (2)	5 (2)	—	0.48
***Herpes*** ** viral infection (H. simplex & H. zoster)**	0	8 (3)	—	0.36
**Bacteremia**	21 (37)	22 (9)	**5.7 (2.7–12.0)**	**<0.001**
**Isolation of multiple organisms in blood**	5 (9)	1 (1)	**22.6 (2.5–522.1)**	**<0.001**
**Bacteriuria**	21 (38)	43 (18)	**2.7 (1.4–5.3)**	**0.003**

*Non-Hodgkin's lymphoma (4 cases), central nervous system tumors (2 cases), and Kaposi's sarcoma (1 case), mediastinal sarcoma (1 case), cervical cancer (1 case), carcinoma of the tongue (1 case) and adenocarcinoma of the colon (1 case).

Figures represent n (%), unless specified. OR: odds ratio. CI: confidence interval. IQR: interquartile range.

Factors independently associated with death included CD4 count ≤200 cells/µl (OR = 16.6, 95% CI = 3.72–74.38), *Pneumocystis jiroveci* pneumonia (OR = 18.5, 95% CI = 4.68–73.26), esophageal candidiasis (OR = 27.5, 95% CI = 5.54–136.90), malignancy (OR = 15.21, 95% CI = 2.33–99.42), and bacteriuria (OR = 7.9, 95% CI = 1.25–50.51). Patients getting ARV prior to admission was found to be a negative predictor (OR = 0.17, 95% CI = 0.06–0.46). [Table 3]

**Table 3:** Independent predictors of death in hospitalized HIV-infected patients.

	Unadjusted		Adjusted	
Predictors	OR (95%CI)	p value	OR (95% CI)	p value
**Nadir CD4 cell count ≤200/mm^3^**	**14.5 (4.4–60.6)**	**<0.001**	16.6 (3.7–74.4)	<0.0001
**Presumptive ** ***Pneumocystis jiroveci*** ** pneumonia**	**10.2 (3.8–28.2)**	**<0.001**	18.5 (4.7–73.3)	<0.0001
**Presumptive osophageal candidiasis**	**6.7 (2.7–16.5)**	**<0.001**	27.5 (5.5–136.9)	<0.0001
**Malignancy**	3.7 (0.9–14.3)	0.04	15.2 (2.3–99.4)	<0.004
**Bacteriuria**	**2.7 (1.4–5.3)**	**0.003**	7.9 (1.2–50.5)	0.02
**Current antiretroviral use**	**0.3 (0.2–0.6)**	**<0.001**	0.2 (0.06–0.46)	<0.001

**Adjustment variables:** New HIV diagnosis, presumptive TB, presumptive histoplasma, bacteremia and isolation of multiple organisms in blood.

OR indicates odds ratio. CI indicates confidence interval.

## Discussion

This report identifies important clinical characteristics and predictors of mortality in hospitalized HIV-infected persons in Bangladesh. Our study demonstrated rates of OIs and inpatient deaths similar to those reported in resource-limited countries with high HIV burden [13], [14]. We also found that most deaths in hospitalized HIV-infected patients are associated with OIs, AIDS-related malignancies, and bacterial infections, similar to reports from other low-income countries [13], [14]. Although available data did not allow us to determine actual causes of death (due to the absence of autopsy facilities and lack of societal acceptance of routine use of autopsies), the high proportions of patients with AIDS-related illnesses who died during hospitalization suggest that most deaths were AIDs-related. This is in contrast to reports from high–income countries, where deaths in HIV-infected patients are now mostly due to non-AIDS related causes [15], [16] and where national screening and early intervention programs are much more widespread.

As expected, nadir CD4 cell count ≤200/mm^3^ was found to be an independent predictor of death, which is similar to findings described in other reports [17], [18]. Individuals hospitalized within six months of HIV diagnosis with were more likely to die during hospitalization. This may be due to diagnosis at an advanced stage of HIV infection. Delays in HIV diagnosis in Bangladesh are likely primarily due to limited access to testing, unwillingness of many to be tested and difficulty identifying individuals who do not have commonly recognized risk factors for infection and thus escaped the routine screening among risk groups [19], [20].

Since the first case of HIV was identified in Bangladesh, the responses and initiatives taken to control and prevent the spread of the disease have included safe needle exchange programs, free condom distribution to sex workers in brothels, safe sex education among peer groups and, recently, the implementation of a methadone program for injection drug users [21]. More than three-quarters of patients in this study had a history of migrant work (Table 1), which has recently been identified as a risk factor for HIV infection in Bangladesh [22], [23]. A recent report from Dhaka Hospital found that among persons diagnosed with HIV infection by provider-initiated HIV Testing and Counselling, 67% of HIV-infected patients had a history of migrant work [24]. Until recently, persons with a history of migrant work were not targeted for HIV prevention interventions as they had not been identified as a group at high-risk for HIV infection [22], [24]. However, it is not known what proportion of HIV-infected persons with history of migrant work were infected abroad or what the risk behaviours of many of those who acquired HIV abroad were. It has been reported that extramarital sex is 2–3 times higher in spouses who live apart, and there is higher proportion of reported sex with female sex workers among male migrant workers [25]. A total of 8% of patients in this study reported injection drug use, compared to 10% of Jagori Ward patients who reported such behaviour in a 2011 report [9].

Consistent with reports from other resource-limited settings, we found that OIs were associated with death among hospitalized HIV-infected persons [14], [20]. Despite a demonstrated reduction of PJP-related mortality after the introduction of ARV medications in other high-income countries [26], [27], our study demonstrated that PJP is an independent predictor of death in HIV-infected patients. In studies from Africa, it has been reported that PJP is the primary cause of death in patients with low CD4 cell counts, despite the availability of prophylaxis against and effective treatment for PJP [17], [28], underscoring the need for early suspicion and treatment of PJP even in the absence of adequate laboratory facilities for confirming the diagnosis.

Our study also showed that having a malignancy was an independent predictor for death. Most malignancies identified were AIDS-related. Our findings are consistent with findings that malignancies are common in HIV-infected individuals, even in those with CD4 cell count >200/mm^3^ [28], and underscore the need for surveillance for malignancies among all HIV-infected individuals.

We found bacteriuria to be independently associated with death. Urinary tract infections occur at increased rates among HIV-infected persons [29], and bacteriuria is more common in those with very low CD4 cell counts [30], which could explain our findings. However, it is also possible bacteriuria is a surrogate marker of disease severity, as urine analyses and urine culture are more commonly performed among those presenting with more serious illnesses.

TB was the most common OI identified among patients in this study, though it was not found to be an independent predictor for death. TB is endemic in the general population in Bangladesh, which may explain why it was not more common among those who died than among those who were discharged in this study. Although we did not find that TB was an independent predictor of death, coinfection with HIV and TB is an important issue in Bangladesh and all persons who are infected with HIV should be screened for active TB.

There are several limitations of this study. First, this was a retrospective, observational study and may have been subject to incorrect interpretation of information. Second, data were missing for some variables and little information available about causes of death. Third, due to laboratory constraints, we typically had to rely on clinical manifestations for OI diagnoses, because confirmatory diagnostic testing results were frequently not available; this could have led to misclassification of some presumptive OI diagnoses and failure to detect OIs in some patients. Finally, this report reflects the experience of just one urban hospital that primarily provides care for patients with late stage HIV disease and so may not be generalizable to HIV-infected patients admitted to other hospitals in Dhaka or elsewhere in Bangladesh.

In conclusion, this study describes factors associated with mortality among hospitalized HIV-infected patients in a country with low HIV prevalence. Most of the factors identified have already been reported in medical literature but original as local data, and could be mitigated or prevented by earlier diagnosis of HIV infection and improved access to care. Expansion of voluntary counselling and testing, and widespread education about HIV and AIDS might lead to increased access to and acceptability of HIV screening. Dissemination of information to providers regarding HIV risk behaviours and clinical features of HIV should also be promoted to increase provider-initiated testing and counselling in healthcare facilities. In addition, it will be critical to increase and improve the training of providers in Bangladesh on HIV care practices, including treatment with ARV medications, prophylaxis against OIs, and the treatment and prevention of other AIDS-related illnesses.
